# Is there a role of synovial biopsy in drug development?

**DOI:** 10.1186/s12891-016-1028-5

**Published:** 2016-04-19

**Authors:** Maria Filkova, Andrew Cope, Tim Mant, James Galloway

**Affiliations:** Academic Department of Rheumatology, Weston Education Centre, King’s College London, Cutcombe Road, SE5 9RJ London, UK; Quintiles Drug Research Unit at Guy’s Hospital, London, UK

**Keywords:** Synovial biopsy, Rheumatoid arthritis, Drug development

## Abstract

Rheumatoid arthritis (RA) is an autoimmune disease which causes significant pain, joint deformity, functional disability. The pathological hallmark of RA is inflammation of the synovium characterized by involvement of inflammatory and resident stromal cells, soluble mediators and signalling pathways leading to irreversible joint destruction. The treatment goal in RA has evolved over the last decade towards a target of disease remission that is achieved in less than a third of patients in clinical trials. The lack of therapeutic response to current treatments is suggestive of alternative drivers of RA pathogenesis that might serve as promising therapeutic targets. There are data to justify the use of synovial tissue in early drug development. Synovial tissue represents an appropriate compartment to be studied in patients with inflammatory arthritis and provides information that is distinct from peripheral blood. Modern techniques have made the procedure much more accessible and ultrasound guided biopsies represent a safe and acceptable option. Advances in analytic technologies allowing transcriptomic level of analysis can provide unique inside to target organ/tissue following the exposure to investigational medicinal product. However, there are still caveats with regard to both the choice of technique and analytical methods. Therefore the significance of synovial biopsy remains to be determined in future clinical trials. The aim of the current debate is to explore the potential for accessing and evaluating synovial tissue in early drug development, to summarize lessons we have learned from clinical trials and to discuss the challenges that have arisen so far.

## Background

Rheumatoid arthritis (RA) is an autoimmune disease, which causes significant pain, joint deformity, functional disability and a significant overall healthcare burden [1]. The treatment goal in RA has evolved over the last decade towards a target of disease remission. Besides conventional synthetic disease modifying anti-rheumatic drugs (DMARDs), biological agents targeting cytokines (TNF-α, IL-1, IL-6) and immune cells (B- and T-lymphocytes) have led to remarkable patient benefits [2]. However, fewer than 30 % of patients in clinical trials achieve disease remission [3].

Although many new agents for treating RA have been evaluated in phase II/III clinical trials in recent years, progression to later phase clinical research or licencing has been limited by concerns about adverse events or lack of therapeutic effect [4]. For example, fostamatinib, a selective inhibitor of spleen tyrosine kinase (SYK), suppressed clinical arthritis and bone erosions in a mouse model of arthritis [5]. However, following four phase II and three phase III clinical trials involving 3200 patients with active RA it was felt that the agent was not worth taking forward to market due to lack of efficacy [4]. The reasons for drugs failing are invariably complex. However, a lack of adequate information about human pharmacodynamics during the early stages of drug development represents a key factor. We hypothesize that early mechanism of action studies with a detailed understanding of the pharmacology of the drug within the target tissue may greatly facilitate progress through clinical development [6, 7].

The pathological hallmark of RA is inflammation of the synovium. This involves a crosstalk between heterogeneous inflammatory and resident stromal cells as well as presence of many soluble mediators and signalling pathways leading to irreversible joint destruction [8]. Given this complexity, the lack of therapeutic response to current treatments is suggestive of alternative drivers of RA pathogenesis that might serve as promising therapeutic targets [9, 10].

**Fig. 1:**
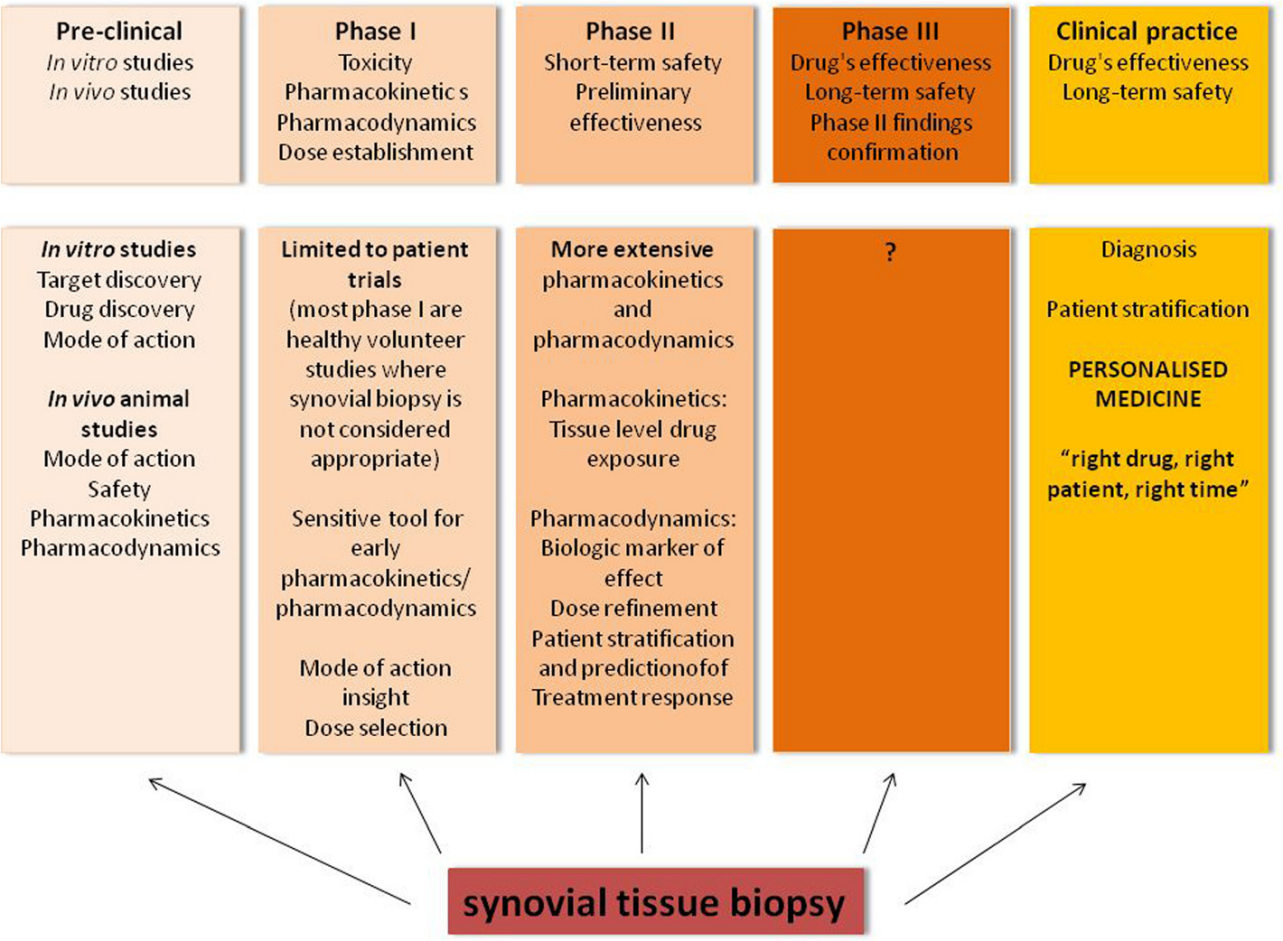
Validated and potential use of synovial tissue biopsy in all stages of drug development and clinical practice

In this debate we aimed to explore the potential for accessing and evaluating synovial tissue in early drug development (Fig. 1), to summarize lessons we have learned from clinical trials and to discuss the challenges that have arisen so far.

## Discussion

### The case for using synovial biopsy in drug development

In phase I clinical trials, safety and tolerability of a new drug is assessed in healthy volunteers adopting a ‘maximum tolerated dose’ approach that seeks to establish drug safety, tolerability, pharmacokinetics and pharmacodynamics of a drug and identify a suitable dose for phase II studies. Phase II looks for signals to support the idea that the drug is efficacious. Early signal of pharmacodynamic effect in target or surrogate tissue may support the idea that the drug has efficacy in patients and may help to establish dosing regimen as shown in oncology trials [7, 11]. Based on phase II outcomes, one or two doses are then tested in phase III trials that will ultimately confirm efficacy in a larger target population and provide the information required for licensure. In addition to time needed for the drug development, it is important to acknowledge that the journey of a drug through these phases costs upwards of £40 million in the current era, with an average drug taking over 7 years to make the transition from phase 1 through to market [12].

Many of the newer agents used for treating rheumatic diseases are monoclonal antibodies (mAb) that have no ‘maximum tolerated dose’. The general approach to selecting a safe human starting dose for mAbs has significantly changed following a series of severe, life threatening adverse events observed during a first-in-human (FIH) clinical trial [13]. The concept of a minimal anticipated biological effect level was coined to provide an understanding of the minimum dose at which pharmacodynamic activity might be anticipated in humans, using data derived from pre-clinical studies in animals or in vitro studies [14].

To complicate matters, the pharmacokinetic properties of a mAb may differ greatly between healthy volunteers and patients with the target disease because the pharmacokinetics may depend upon the levels of target ligand present and the role of the target molecule in normal and pathophysiological condition [15, 16]. Healthy volunteers may express the therapeutic target to much lesser extent than the patients or may not express it at all. Therefore choosing doses for subsequent study is more challenging as too high a dose may lead not only to early toxicity, but also the delayed effects of immune suppression (e.g. infection or cancer). Conversely, selecting too low a dose may lead to a failure to demonstrate efficacy and result in inappropriately stopping further study of a potentially useful agent. For example, levels of IL-17, which contributes to the pathogenesis of RA, are extremely low or undetectable in healthy humans while IL-17 is elevated in sera and synovial fluid of patients with RA and IL-17 is present at site of inflammation in RA synovial tissue [15, 17]. Therefore, an FIH trial of an anti–interleukin-17 monoclonal antibody performed in patients with RA instead of healthy volunteers was a logical approach [18].

Pharmacodynamic effects of investigational medicinal product (IMP) in RA can be analyzed either in peripheral blood (plasma/serum/blood cells) or, with respect to RA pathogenesis, in synovial tissue. Studies have shown that expression of biomarkers in synovial tissue may be independent of peripheral blood [19, 20].

Natural biological variability in disease activity and synovial tissue could be an important aspect in long-term trials. In comparison to the variations observed in clinical assessment, laboratory assessment including synovial tissue changes are more stable and less susceptible to placebo response [21–23]. Analysis of synovial tissue during clinical trials appears a sensible approach to document changes directly at the site of inflammation and joint destruction. Published analysis of synovium has been mostly limited to histology or single gene transcript [24, 25]. These approaches, although providing clinically useful information, were not granular enough to inform early phase trial development. Studies of synovial biopsies using state-of-the-art technologies and composite ontogenetic/pathway analysis of gene clusters have been shown to be more informative and relevant when compared to analysis of individual markers [20, 26].

Finally, clinical outcomes require longer time follow up and larger patient numbers that are permitted in early phase studies [27]. Therefore there is an urgent need to better quantify drug effect using alternative approaches in small proof-of-principle studies [6]. The earlier on in clinical development a signal of pharmacodynamics effect can be identified, fewer unnecessary people will be recruited to trials, and novel drugs will have a higher chance of succeeding through later phases.

Considering that synovial hyperplasia and its aggressive potential are the hallmark of RA, analysis of early pharmacodynamic effects of IMP in synovial tissue in early phase clinical trials may be of great interest.

### Synovial biopsy technique and safety – new approach on board

In order to obtain synovial tissue from affected joint, either an open method using surgical intervention or minimally invasive techniques may be used. The latter have been increasingly utilised due to minimal burden on patients’ well being [24, 28].

A widely used method, a gold standard of synovial tissue biopsy enabling visualisation of the joint, is to acquire tissue at arthroscopy [27]. Although arthroscopic biopsies are usually logistically more challenging, as they require theatre settings and tend to be performed on larger joints, smaller procedure rooms or the outpatient clinic setting appeared feasible and accessible to rheumatologists [29]. The knee is the commonest site of arthroscopy, although other smaller joints are accessible to arthroscopy when an arthroscope of smaller diameter is used. This is essential at early stages of the disease when predominantly hand and wrist joints are affected. An important advantage of arthroscopy is direct visualisation of the examined joint enabling macroscopic evaluation of synovial tissue and cartilage and obtaining adequate amount of tissue for further sample processing [24].

Development and widespread use of ultrasound by rheumatologists has supported the use of ultrasound-guided synovial biopsies, merging the minimally invasive needle biopsy with the advantage of visualized guidance [30]. Although concerns about the validity of tissue sampling for further sample processing and use other than immunohistochemistry (e.g. molecular biology) have been raised in the past [27], current knowledge provides a firm evidence of good safety profile, tolerability and reproducibility of high quality of sample collection even from the small joints that are most frequently affected joints in RA [30]. While previous studies obtained 6–8 samples per procedure, retrieving up to 12–15 samples was shown to be tolerated by patients [28, 30].

In summary, recent advances in imaging, minimally invasive biopsy procedures and tissue analysis offer a unique opportunity to use synovial tissue in early drug development given the discovery of new drivers and novel therapies in RA. Learning from the oncologists’ approach of performing whole genome transcriptomics on very small samples of tissue in proof-of-concept studies [31], there is the potential to adopt this approach in RA.

### Obtaining high quality tissue and potential pitfalls

A major challenge of synovial tissue biopsies is obtaining representative samples suitable for further analysis. Several pitfalls during the process of tissue biopsy including selection of site of biopsy deserve consideration.

First, it is essential to identify a joint with inflamed synovium for biopsy. RA is a polyarticular disease affecting predominantly hand joints. However, these may not often be suitable for synovial biopsy despite availability of minimally invasive techniques. Therefore the question arises as to whether there are differences between the synovium in the large and small joints. In general, cell numbers present in synovial biopsies from knee joint or small joint are comparable with a strong correlation for the number of macrophages, T cells and plasma cells along with IL-6 expression in the sublining layer across joints. These results suggest that the inflammation in one inflamed joint is generally representative of that in other inflamed joints from the same patient. In contrast, hyperplasia of intimal lining appears to depend on local processes as the numbers of intimal macrophages or synovial fibroblasts in the lining layer do not correlate between the different joints [32].

However, data obtained from open procedures (e.g. joint replacement surgery) have revealed that significant tissue heterogeneity is apparent within a single joint. It was shown that number of differentially expressed genes between biopsies from the same patient was about three times larger in orthopaedic than in arthroscopic biopsies because open tissue biopsy included heterogeneous quality samples from both inflamed and non-inflamed regions [33]. Arthroscopic biopsies do allow direct visualisation of the joint and therefore permit a targeted biopsy of affected tissue, although despite this, arthroscopic biopsies still do not guarantee adequate tissue for analysis [24, 34].

Ultrasound has a distinct advantage in the identification of synovial tissue for biopsy by allowing assessment of both synovial thickening and tissue vascularity, which can be considered a surrogate for the direct visualisation of inflamed tissue. Pre-biopsy grey-scale US assessment of synovitis predicts synovial tissue quantity/quality for histological and RNA analyses in contrast to power Doppler [30].

Several studies analyzed histological variability between multiple specimens obtained during one biopsy procedure. Histological analysis of tissue specimens obtained from different areas the knee joint, including those of apparent maximal and minimal involvement, showed wide range of histological scores that reflects natural biological variability [34, 35]. Other studies showed similar pattern of cell infiltrates and expression of selected mediators at mRNA or protein level when compared synovial tissue originating from the junction or suprapatellar pouch [36, 37]. However, it is preferential to obtain sections derived from multiple sites within one joint for analysis based on natural variation within synovial tissue [38, 39].

### Synovial tissue analysis in trials – which method is the best?

**Fig. 2:**
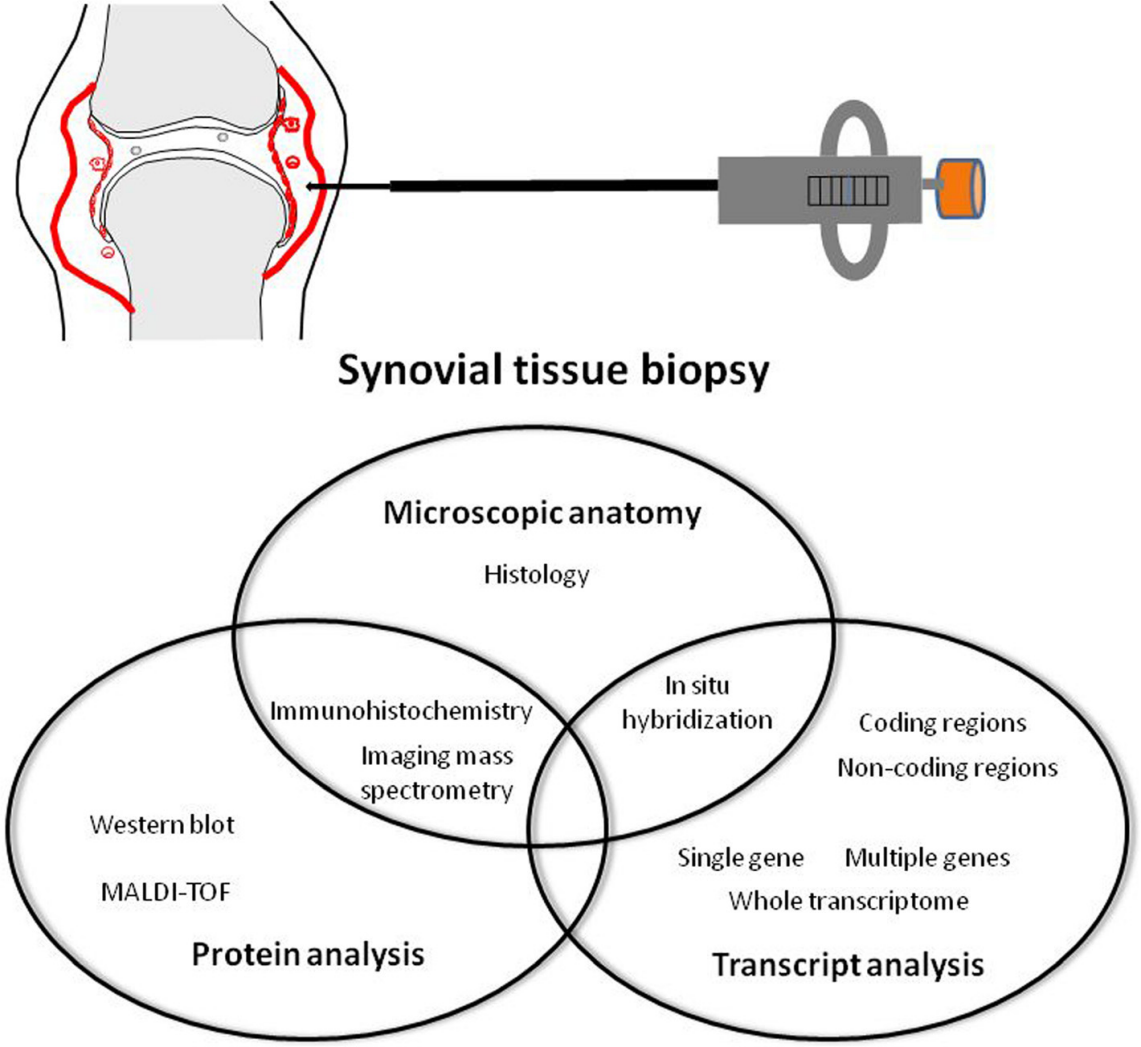
Methods of synovial tissue analysis with implications for drug development

As summarized above, the optimal approach to obtain representative samples is to directly visualize inflamed tissue arthroscopically or with ultrasound. Direct visualization during biopsy minimizes sampling error, enables acquisition of biopsies from precise locations within joints and provides reproducible good quality tissues. Available techniques for synovial tissue analysis will now be summarized (Fig. 2).

#### Histological analysis and immunohistochemistry (IHC)

The typical feature of RA synovium is synovial thickening in the lining layer due to infiltration of monocytes/macrophages and excessive proliferation of synovial fibroblasts, in the sublining layer by marked cellular infiltrate, which includes synovial fibroblasts, macrophages, CD4+ and CD8+ T cells, B cells, plasma cells, dendritic and mast cells, in addition to increased vascularity due to enhanced angiogenesis [40]. Immune cells can be grouped into follicular structures (follicular synovitis, mostly B cells) in 30–40 % of patients or be randomly distributed within the sublining layer (diffuse synovitis, predominantly CD68+ cells). In contrast, another type known as pauci-immune synovitis shows hardly any infiltrating immune-cells and may be present in active disease [41].

Histology and IHC enable visualization of cell/protein markers expression and distribution in synovial tissue. An accurate estimate of the overall joint should be satisfactorily provided by analysis of cumulative area of 2.5 mm^2^ from at least 3 biopsy specimens when sample is obtained during arthroplasty, arthroscopy or blind synovial tissue biopsy from small or large joints [42, 43]. However, 6–8 tissue samples are usually obtained for further analysis [28, 30]. US-guided biopsy was shown to approach 92.5–100 % success in acquisition of histologically reliable tissues [30, 42].

Methods used for quantitative analysis of IHC data have both advantages and limitations [24, 38, 39]. One potential error may occur due to lack of internal normalization or non-specific accumulation of pigmented precipitate detected by enzymatic processes [39]. Although manual cell counting is considered a standard histological analysis, it is time consuming and faces several other pitfalls, e.g. observer bias, limited number and selection of fields to be analyzed that may cause significant limitation in large clinical trials. Widely used semi-quantitative analysis and latest techniques using computer based digital image analysis may minimize these errors, are acceptable for whole tissue analysis of widespread targets, lack field selection errors and bias may be minimized by using grading scales and analyses performed by independent readers.

Semi-quantitative histopathological scoring system (from 0, absent to 3, strong) was used to evaluate 3 features of chronic synovitis: enlargement of lining cell layer, cellular density of synovial stroma and leukocytic infiltrate. The sum provided the synovitis score (0–1, no synovitis; 2–4, low-grade synovitis; 5–9, high-grade synovitis) that showed 61.7 % sensitivity and 96.1 % specificity for RA-related high-grade synovitis and contributed to the diagnostics of rheumatic and non-rheumatic joint diseases [44].

#### Western blot and mass spectrometry

The western blot is an analytical technique used to detect specific proteins of interest in tissue homogenate or extract but has limited value in clinical trials. Instead, analyses using mass spectrometry may become a promising method to analyze proteome from whole tissue extracts. Matrix assisted laser desorption/ionization combined with time-of-flight detection (MALDI-TOF) was recently shown to distinguish different subgroups of juvenile idiopathic arthritis [45]. Imaging mass spectrometry that combines morphology and mass spectrometry has been used to illustrate various mass spectra generated from proteins expressed in RA and osteoarthritis synovial tissue [46]. In theory MALDI-TOF also offers an opportunity to quantitatively assess tissue level drug exposure directly, although this has not previously been done. Mass cytometry, previously used for cell suspensions, has recently been coupled with IHC and immunocytochemical methods with high-resolution laser ablation to CyTOF mass cytometry [47]. Although this method has only been applied to malignant tissue so far, it is a candidate approach that could identify over 100 protein markers in conjunction with a CyTOF machine enabling high-dimensional, single-cell analysis of cell type and state, cell interactions and tissue structure in future targeted diagnosis and therapies [47].

#### In situ hybridization

Similar to IHC, in situ hybridization shows a target of interest at the transcriptional level (messenger RNA, mRNA) and enables visualisation of mRNA distribution within tissue. Information obtained by in situ hybridization can be reliably confirmed at the protein level by IHC [48]. Albeit that this method is powerful enough to detect even small non-coding RNAs such as miRNAs, it is time consuming, requires multiple steps to be optimized for every single target or tissue and, like IHC, enables detection of only limited number of genes.

#### Single/multiple gene analysis and transcriptome profiling

Gene expression technology has expanded enormously in recent years and enables analysis of single genes, groups of genes or whole genome wide expression profiles including protein-coding or non-coding regions, although sufficient tissue is needed for RNA extraction. Needle arthroscopy using 2-mm grasping forceps and obtaining 6 biopsy specimens provided at least 15–50 mg for further RNA analysis [49]. Other studies using needle arthroscopy approach showed that at least 4 biopsy samples provided 1–2 μg of total RNA [50, 51]. Yield from ultrasound guided synovial tissue biopsy taking 6 samples with 16/14G quick core needles provided at least 10 mg of synovial tissue for RNA isolation, with a median RNA yield 0.54–0.89 μg/10 mg tissue. The yield varied according to joint size with a tendency for lower yields obtained from small joints [30]. General, > 1 ug of RNA is needed for transcriptomic studies, particularly RNA-sequencing [52], which is generally at the threshold of total RNA obtained from synovial tissue biopsies. A relatively new approach to overcome this issue is a low-input 5’- or 3’-RNA-sequencing, where the terminal 300–500 bp of either the 5’- or 3’-end of the mRNA is sequenced instead of the entire transcript and low quantities of starting material (as low as 1 ng total RNA) can be used [53].

Considering serial tissue biopsies for clinical trial purposes, similar numbers of biopsies as well as RNA yield was obtained in subsequent biopsies 12 weeks apart and demonstrated evidence of tissue level pharmacodynamic response [30]. These data suggest that ultrasound-guided synovial tissue biopsies have the potential to be taken forward as a method of choice in the experimental setting associated with drug development.

In keeping with sampling errors discussed above, transcriptome profiling also carries a risk of error due to biological or technical variability [54]. The manner by which these factors influence gene expression profiles remains unsure as changes in transcriptome can occur very rapidly: while one study emphasized the major impact of biological variability (age, sex, time of the day, cellular composition), another study showed that technical variability is of greater importance [54, 55]. Also, intra-patient variations in biopsies are smaller than inter-patient variations, reflecting unique mRNA signatures of each patient rather than tissue heterogeneity [56].

Microarray technology, that uses selected probes placed on solid matrix, is limited by the reliance upon existing knowledge about genome sequence, high background noise and a limited dynamic range [31]. In contrast, RNA sequencing enables studying the whole transcriptome including gene boundaries and introns, splicing diversity as well as detection of small RNAs in a high-throughput and quantitative manner [57].

#### Transcriptome interpretation

Studies using state-of-the-art technologies and composite ontogenetic/pathway analysis s have been shown to be more informative and relevant compared to analysis of individual markers [24–26]. Such comprehensive analysis in tissue/blood may indentify single genes or gene combinations/subsets that may have a predictive/diagnostic value and may be taken forward for further validation [58–60]. For example, a set of 20 genes selected from microarray analysis of white blood cells had 90 % sensitivity and 70 % specificity whilst a combination of only 8 genes had 80 % sensitivity and 100 % specificity to distinguish RA responders or non-responders to infliximab [58]. However, expression profiles, providing e.g. a gene expression signature predictive of therapeutic response, are often inconsistent across studies [59]. Protocol standardization from preanalytical phase until data analysis is therefore crucial for obtaining reliable, consistent and reproducible data [25, 27]. Also, selection of the same tissue type, patients and clinical parameters are important to be considered when different studies are compared [61]. Importantly, genome-wide expression profiling, changes in transcriptome, pathway/ontology analysis, and comparison between different cell types/compartments may unravel complex molecular interactions and reflect responses to therapy.

### What have we learned from histopathological analysis of synovial biopsies in clinical trials?

As mentioned above, the increasing pressure to obtain accurate and reliable outcome data in early phase clinical trials draws attention to synovial biopsies. Recognition of markers of early drug response at synovial tissue level would be of great value in proof-of-concept studies and would help to make early go/no go decisions on progression of the drug to next phase level. Also, multiple studies using IHC have revealed association between baseline synovial tissue biomarkers, their change upon therapeutic intervention and future disease response. Much of the data were generated after exposure to very well known, newly introduced and experimental drugs, and are summarized in Table 1. The overview is based on a complex literature review providing results of original studies and summaries of systematic reviews.

**Table 1:** Overview of immunohistopathological data obtained from small proof-of-concept studies using synovial biopsies in RA

Drug	Timing of biopsies	Key finding	Reference
DMARDs/anti-TNF/experimental	2–16 weeks	Number of CD68+ macrophages in sublining synovial layer is a good biomarker of therapeutic response.	[76–79]
Methotrexate	16 weeks	Decrease in the numbers of inflammatory cells, including CD3+ and CD8+ T cells, CD38+ plasma cells, CD68+ macrophages (lining layer), inflammatory and destructive mediators (Ki67, IL-1β, TNF-α, E-selectin, ICAM-1, VCAM-1, MMP-1). Responders displayed a reduction in the expression of all ICAM-1, VCAM-1, TNF-α and IL-1 β.	[80, 81]
Methotrexate	12 weeks	No change in synovial hyperplasia, lymphoplasmocytic infiltrate, CD68+ macrophages, CD3+ T cells and CD138+ plasma cells	[50]
Leflunomide	16 weeks	Decrease in the numbers CD68+ macrophages (sublining), inflammatory and destructive mediators (ICAM-1, VCAM-1, MMP-1). Responders displayed a reduction in the expression of ICAM-1, VCAM-1 and TNF-α.	[81]
Prednisolone	24 h	Decrease in the expression of TNF-α (lining and sublining), IL-8 (lining), as well as reduced synovial fluid IL-8 levels. Change in TNF-α correlated with clinical response to, and subsequent relapse after therapy	[82]
Prednisolone	2 weeks	Reduction in the number of sublining synovial macrophages, a trend toward decreased infiltration by CD4+ T cells, CD38+ plasma cells, and CD55+ fibroblast-like synoviocytes	[76]
Infliximab (3 mg/kg)	48 h/4 weeks	Reduced number of CD68+ intimal macrophages after 48 h, a trend to decreased amount of CD38+ plasma cells, CD3+ T cells, sublining CD68+ macrophages after 48 h/4 weeks. Decreased numbers of CD3+, CD38+ and both intinal and sublining CD68+ cells were observed in clinical responders after 4 weeks.	[23]
Infliximab (10 mg/kg)	2 weeks	Reduction in the numbers of infiltrating synovial CD3+ T cells, CD22+ B cells, CD68+ macrophages and in the expression of IL-8, MCP-1 and TNF-α. High levels of synovial TNF-α prior to treatment may predict responsiveness to therapy.	[83, 84]
Rituximab	4 weeks	Incomplete depletion of CD22+ B cells, no correlation with the change in DAS28.	[62]
Rituximab	4 weeks 16 weeks	CD19+ B cells significantly but incompletely decreased at 4 weeks, with further reduction at 16 weeks in some patients. Decrease in CD68+ macrophages at 4 and 16 weeks, CD3+ T cells decreased at 16 weeks. The reduction of CD138+ plasma cells predicted clinical improvement at 24 weeks.	[64]
Rituximab	12 weeks	Depletion of CD20+ B cells, trend to decrease in CD68+ macrophages. No correlations between changes in CD20+ or CD68+ and changes in the DAS28. Positivity for circulating IgM ACPA, in combination with a high infiltration of CD79a + B cells is a predictor of clinical outcome after rituximab.	[51, 65]
Tocilizumab	12 weeks	Decrease in synovial hyperplasia, lymphoplasmocytic infiltrate, CD68+ macrophages, CD3+ T cells and CD138+ plasma cells	[50]
Anakinra + pegsunercept	4 weeks 52 weeks	Decrease in number of CD3+ T cells and TGFβ expression as biomarker therapeutic response at weeks 4 and 52 of combination therapy. Baseline CD3+ and sublining CD68+ infiltration, VEGF and TGFβ expression were predictive of subsequent structural outcome at 6 or 12 months.	[19]
CCR1 antagonist	2 weeks	Reduction in overall cellularity, number of CD4+ and CD8+ T cells, CD68+ macrophages and the number of CCR1+ cells.	[22]
RecIL-10	12 weeks	No significant change in number of inflammatory cells or in the scores for the expression of cytokines.	[85]
IL-1 receptor antagonist	24 weeks	Reduction in intimal and sublining CD68+ macrophages and CD3+ lymphocytes.	[86]
Anti-CCL2 antibody	6 weeks	No immunohistologic changes.	[66]
C5aR-antagonis	4 weeks	No immunohistologic changes.	[67]
IFN-β (18/36/54 million IU/week)	4 weeks 12 weeks	Decrease in number of CD3+ T cells at 4 weeks and CD38+ plasma cells at 12 weeks along with changes of several inflammatory and destructive molecules (e.g. MMP-1, IL-6 or IL-1β).	[87]
IFN-β (6.6/132 μg/week)	24 weeks	No changes in synovial tissue infiltrates.	[68]

These data must be compared and interpreted with caution given the different timelines, disease characteristics of patient groups and immunolabeling, e.g. B cell markers CD19, CD22 and CD20 that are not jointly expressed during the maturation of B cells [62–65]. As some data are in contrast to changes documented in peripheral blood, composition of synovial tissue infiltrates and expression of mediators may drive local inflammation or be associated with early relapse of RA and underscore the importance of looking at synovial tissue [62, 64]. Also, the absence of clinical effect of a study drug/placebo is accompanied by the lack of changes in sequential biopsies [21, 66–68]. This supports the importance of synovial tissue analysis during treatment that may be more sensitive than clinical assessment.

### Synovial transcriptome: a new biomarker?

Data on complex gene expression analysis on RA, osteoarthritis and healthy synovial tissue suggested that this approach could be taken forward to test transcriptome responses following treatment at synovial tissue level [69, 70]. Both immune and stromal cells contribute to tissue heterogeneity resulting in clusters of differentially expressed genes in keeping with different pathophysiologic processes across patients [20, 41, 71]. A group of RA patients with high inflammatory gene expression pattern indicated involvement of immunity and defence pathways and activation of B and T cells while the other group showed predominantly involvement of cell-communication, developmental and fibroblast-dedifferentiation pathways [9, 20, 41]. Similarly, gene expression in synovial tissue from patients with early RA identified at least two patterns of synovitis, one of which resembled long-standing RA [72]. In addition, distinct baseline synovial gene expression signature reflecting phenotypes of RA synovium (lymphoid, myeloid, low inflammatory, fibroid) may be predictive of treatment response: synovial myeloid, but not lymphoid, gene signature expression was higher in patients with good compared with poor response to anti-TNFα therapy [71]. These data suggest that RA patients with different pathway activation may need different therapeutic approaches. Examples of comprehensive gene expression analysis aiming for individualized therapy and understanding of mode of action of novel therapies are summarized in Table 2.

**Table 2:** Overview of gene expression analysis obtained from small proof-of-concept studies using synovial biopsies in RA

Drug	Timing of biopsies	Key finding	Reference
Infliximab	Baseline	Differential baseline gene expression in responders and non-responders. Overexpression of genes involved in T-cell mediated immunity, cell surface receptor mediated signal transduction, major histocompatibility complex II (MHCII)-mediated immunity, cell adhesion, cytokine and chemokine mediated signalling, cell adhesion mediated signalling, signal transduction, and macrophage-mediated immunity identified in responders.	[88]
Infliximab	9 weeks	Unique baseline transcriptome in all patients. 279 differentially expressed genes between good responders and non-responders. Significant change in expression of 115 genes in the good responding group involved in immune response, cell communication, signal transduction and chemotaxis.	[56]
Adalimumab	12 weeks	Deregulated baseline expression of 439 genes involved in cell cycle and immune responses in good vs. poor responders. Differential expression of 632 genes enrolled in cell division, signal transduction, antigen processing/presentation, T-cell activation, and apoptosis upon adalimumab treatment in a group of good responders.	[73]
Rituximab	12 weeks	Deregulated baseline expression of 2458 genes involved in immunoglobulin clusters, antigen processing and presentation via MHCII in non-responders vs. responders. Treatment with rituximab resulted in downregulation of 220 genes enriched in immunoglobulin clusters, chemotaxis, leukocyte activation and immune responses; upregulation of 329 genes involved in cell development and wound healing.	[51]
Rituximab	12 weeks 21 months	Baseline differential expression of genes involved in T cell and macrophage function, remodelling and interferon-α biology between non-responders vs. responders at months 3, 9 and 21. Downregulation of CD20 at 3 and 12 months, differential expression of multiple genes involved in B and T cell biology at 21 months (e.g. CD27, CD38, CD8, CD52, CTLA4, CD122, FOXP3, IL-6, IL-12, IL-13, IL-17RA, IL-23a, IL-32, CCL5, MMP3, FASLG)	[89]
Tocilizumab	12 weeks	Downregulation of 3413 genes involved in cytokine/chemokine pathways and T cell activation, upregulation of 3270 genes involved in healing process. Downregulation of genes involved in induction of apoptosis and myeloid cell differentiation, and upregulation of genes involved in regulation of Ras protein signal transduction and ubiquitin-dependent protein catabolic processes observed in responders achieving remission at 6 months.	[50]
Methotrexate	12 weeks	Downregulation of 586 genes enriched in T cell activation and immune response pathways, upregulation of 610 genes. Downregulation of genes enrolled in cell division in responders achieving remission at 6 months.	[50]

Importantly, the differences between study designs and selection of patients should be considered. For example, treatment with methotrexate, tocilizumab and rituximab had similar molecular effects on transcriptomic changes (albeit of different magnitudes) in the RA synovium, which were distinct from the molecular changes induced by adalimumab [50, 51, 73]. It is important to notice that patients included in these trials were at different stages of the disease, since patients on tocilizumab or methotrexate were treatment naive, patients on adalimumab had failed DMARD therapy and patients commencing rituximab were both DMARDs anti-TNF failures. Although to date transcriptome analysis has been directed towards prediction of clinical response, its greatest strength may lie in helping understand of drug mode of action.

### Is transcriptome analysis in peripheral blood superior to synovial tissue?

Compared to synovial biopsy, obtaining peripheral blood to explore new biomarkers of treatment response may be convenient due to the quick and non-invasive approach. However, there is evidence that transcriptome analysis in peripheral blood and synovial tissue may be of different value:

While gene expression profiling in RA synovial tissue mentioned above [20] revealed 2 groups of patients based on differential involvement of molecular pathways in keeping with the immunohistopathological picture, gene expression profiles in peripheral blood did not reflect the differential tissue pathology.

Gene expression analysis using a microarray approach was performed on RNA from peripheral blood mononuclear cells (PBMC) 72 h after a single dose of etanercept [74]. Although a combination of 46 genes at baseline level did not predict therapeutic response, the change in their expression was associated with disease activity at 3 months. Pathway analysis in PBMC revealed TNFα and IL-6 related downstream changes in responders while TNF-α related mechanisms were detected in non-responders suggesting differential regulation in non-responders [74]. Also, infliximab dampened immune responses in PBMC one month after first dose [75]. It was shown that all RA patients have a similar magnitude of down-regulation of genes involved in inflammation, angiogenesis and T and B cell activation irrespective of clinical response evaluated after 16 weeks, highlighting that the transcript changes observed in peripheral blood correlate less well with clinical response compared to synovium.

## Conclusions

There is grooving evidence to justify the use of synovial tissue in early drug development. Synovial tissue represents an appropriate compartment to be studied in patients with inflammatory arthritis and provides information that is distinct from peripheral blood in the context of RA. Modern techniques have made the procedure much more accessible and ultrasound guided biopsies represent a safe and acceptable option. Advances in analytic technologies allowing transcriptomic level of analysis can provide unique inside to target organ/tissue following the exposure to IMP. However, there are still caveats with regard to both the choice of technique and analytical methods. Therefore the significance of synovial biopsy remains to be determined in future clinical trials.

### Ethics and consent to participate

Not Applicable.

### Consent to publish

Not Applicable.

### Availability of data and materials

The authors don’t provide any original data. All references supporting authors’ conclusions are addressed within the manuscript.

## Abbreviations

CyTOF: Cytometry by Time of Flight
DMARDs: disease modifying anti-rheumatic drugs
FIH: first-in-human trial
IFN: interferon
IHC: immunohistochemistry
IL: interleukin
IMP: investigational medicinal product
mAb: monoclonal antibody
PBMC: peripheral blood mononuclear cells
MALDI-TOF: matrix assisted laser desorption/ionization combined with time-of-flight detection
RA: rheumatoid arthritis
RNA: ribonucleic acid
